# Early-life exposure to PM_2.5_ and risk of acute asthma clinical encounters among children in Massachusetts: a case-crossover analysis

**DOI:** 10.1186/s12940-018-0361-6

**Published:** 2018-02-21

**Authors:** Roxana Khalili, Scott M. Bartell, Xuefei Hu, Yang Liu, Howard H. Chang, Candice Belanoff, Matthew J. Strickland, Verónica M. Vieira

**Affiliations:** 1 Environmental Health Sciences Graduate Program, Susan and Henry Samueli College of Health Sciences, University of California, Irvine, CA USA; 2 Program in Public Health, Susan and Henry Samueli College of Health Sciences, University of California, Irvine, 653 E. Peltason Dr., AIRB 2042, Irvine, CA 92697-3957 USA; 3 Department of Statistics, Donald Bren School of Information and Computer Sciences, University of California, Irvine, CA USA; 4 Department of Epidemiology, School of Medicine, Susan and Henry Samueli College of Health Sciences, University of California, Irvine, Irvine, CA USA; 5 Department of Environmental Health, Emory University Rollins School of Public Health, Atlanta, GA USA; 6 Department of Community Health Sciences, Boston University School of Public Health, Boston, MA USA; 7 School of Community Health Sciences, University of Nevada, Reno, NV USA

**Keywords:** Asthma, Child, Particulate matter, Low birthweight, Case-crossover

## Abstract

**Background:**

Associations between ambient particulate matter < 2.5 μm (PM_2.5_) and asthma morbidity have been suggested in previous epidemiologic studies but results are inconsistent for areas with lower PM_2.5_ levels. We estimated the associations between early-life short-term PM_2.5_ exposure and the risk of asthma or wheeze clinical encounters among Massachusetts children in the innovative Pregnancy to Early Life Longitudinal (PELL) cohort data linkage system.

**Methods:**

We used a semi-bidirectional case-crossover study design with short-term exposure lags for asthma exacerbation using data from the PELL system. Cases included children up to 9 years of age who had a hospitalization, observational stay, or emergency department visit for asthma or wheeze between January 2001 and September 2009 (*n* = 33,387). Daily PM_2.5_ concentrations were estimated at a 4-km resolution using satellite remote sensing, land use, and meteorological data. We applied conditional logistic regression models to estimate adjusted odds ratios (ORs) and 95% confidence intervals (CI). We also stratified by potential effect modifiers.

**Results:**

The median PM_2.5_ concentration among participants was 7.8 μg/m^3^ with an interquartile range of 5.9 μg/m^3^. Overall, associations between PM_2.5_ exposure and asthma clinical encounters among children at lags 0, 1 and 2 were close to the null value of OR = 1.0. Evidence of effect modification was observed by birthweight for lags 0, 1 and 2 (*p* < 0.05), and season of clinical encounter for lags 0 and 1 (*p* < 0.05). Children with low birthweight (LBW) (< 2500 g) had increased odds of having an asthma clinical encounter due to higher PM_2.5_ exposure for lag 1 (OR: 1.08 per interquartile range (IQR) increase in PM_2.5_; 95% CI: 1.01, 1.15).

**Conclusion:**

Asthma or wheeze exacerbations among LBW children were associated with short-term increases in PM_2.5_ concentrations at low levels in Massachusetts.

**Electronic supplementary material:**

The online version of this article (10.1186/s12940-018-0361-6) contains supplementary material, which is available to authorized users.

## Background

Asthma is a common chronic lung disease in which the airways become inflamed and produce increased mucus, making air flow in and out of the lungs difficult. The prevalence of asthma is higher among children 0–17 years of age compared to adults 18 years of age or older [1, 2]. Asthma is typically diagnosed starting at age 5; however symptoms may appear at younger ages. Children under the age of 5 may be more likely to have asthma if they have airborne allergies, wheeze without a cold, and if the parents have been diagnosed with asthma [2]. In addition to family history, children who had previously acquired respiratory syncytial virus (RSV), the virus primarily detected among infants diagnosed with viral bronchiolitis, were more likely to develop wheeze or asthma throughout both childhood and adulthood [3–5].

Several environmental exposures have suggested to increase the risk of asthma exacerbation, including both indoor and outdoor air quality [6–11]. Ambient air pollutants, including fine particulate matter (PM_2.5_), have been shown to play a role in adverse respiratory outcomes among children with asthma [12]. PM_2.5_ represents a combination of small particles and liquid droplets from vehicular exhaust, coal and wood burning, and industrial activities.

Measurements of residential distance to major roads and traffic density have been used as indirect estimates of traffic-related air pollutants due to the difficulty of measuring the mixture of traffic-related pollutants [13, 14]. Adverse respiratory outcomes have also been associated with exposure to areas of high traffic density or near major roadways [15–19]. Concentrations of vehicular pollutants have shown to decline to background levels after 150–300 m (m), while asthma prevalence has been reported to be higher among children who live closer to major roads [16, 17, 20]. The effects of air pollution in both warm and cool seasons have been associated with pediatric asthma attacks [21–24].

Although adverse health effects of ambient PM_2.5_ exposure have been shown across all populations, infants and young children are sensitive subgroups. The small size of PM_2.5_ makes the particles easy to inhale and penetrate deep into the airways [25]. In children, the immune and respiratory systems are not fully developed and their time spent outdoors is greater than adults. Young children also breathe more air per body weight than adults which in turn makes them more susceptible to ambient air pollutant exposures [26].

Notwithstanding some studies associating air pollution exposure and childhood asthma development and exacerbation, it is unclear whether lower levels of PM_2.5_ trigger the same responses [27–29]. In addition, many large population-based studies have not studied effect modification between asthma exacerbations and short-term changes in PM_2.5_ among potentially susceptible subgroups due to the lack of data. The aims of the study were to investigate the associations between early-life short-term PM_2.5_ exposure and the risk of asthma or wheeze exacerbation, and to determine if there was any effect modification. To do this, we employed a case-crossover study design with short-term exposure lags using data on clinical encounters among all children 0–9 years of age in Massachusetts (MA) obtained from the innovative pregnancy to Early Life Longitudinal (PELL_ cohort record linkage system.

## Methods

### Study population

**Fig. 1:**
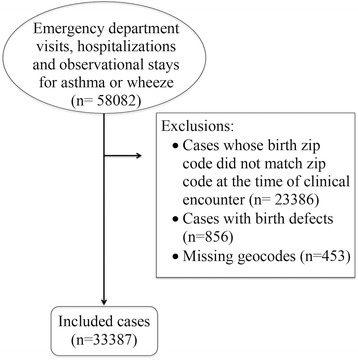
Flow chart of study population

The PELL linkage system links all MA births to administrative databases including hospitalizations, emergency department visits, and observational stays. Pediatric emergency department, hospitalizations, and observational stay data were combined and included in a single analysis because of the similarity of cases. The case definition for asthma or wheeze included the first clinical encounter for children from 0 to 9 years of age using all International Classification of Diseases, Ninth Revision (ICD-9) diagnosis fields (ICD-9 code 493 or 786.07) between January 2001 and September 2009. Our data included street-level birth addresses and zip codes at time of clinical encounter. We excluded children who did not have the same zip code at the time of their clinical encounter and at birth because the PM_2.5_ concentration at their residence at birth was unlikely to be representative of their PM_2.5_ exposure at the time of their clinical encounter. We also excluded any children whose birth address was unable to be geocoded and children with birth defects. The final number of participants included in our analysis was 33,387 (see Fig. 1). After PM_2.5_ exposures were assigned, approximately 10% of our study population had missing PM_2.5_ estimates and were not included in the analysis. A secondary analysis using only primary and secondary ICD-9 fields was performed to account for any diagnosis misclassification that may result from using all ICD-9 fields (*n* = 30,301). Because asthma is typically best diagnosed at age 5 or older, a sensitivity analysis was conducted with cases age 5 and older (*n* = 6195).

### Exposure assessment

PM_2.5_ concentrations were modeled spatiotemporally using satellite remote sensing, meteorological and land use data for the same duration as the study period. Daily PM_2.5_ concentrations in MA were estimated for a 4-km (km) spatial grid from a three-stage statistical model using aerosol optical depth (AOD) data from the Geostationary Operational Environmental Satellite (GOES). The first stage included a linear mixed effects (LME) model accounting for temporal variation between PM_2.5_ and satellite based AOD by using day-specific random intercepts and slopes for AOD and meteorological data [30, 31]. The second stage accounted for spatial variability between PM_2.5_ and AOD using a geographically weighted regression (GWR) [32, 33]. Lastly, the third stage used the previously predicted PM_2.5_ concentrations across the study area from stages 1 and 2 to model areas in the study grid with missing AOD data [34]. However, if AOD data was completely missing for one day then no predictions were made from any stage of the model.

Distance to road (in meters) was estimated using geographic information systems (GIS) for all geocoded births. Road segments were obtained from the MA Department of transportation (MassDOT) roads. Major Roads (CLASS = 1–4) were used in the analysis and include limited access highways, multi-lane highways, numbered routes, and major roads.

### Statistical analysis

Odds ratios (OR) and 95% confidence intervals (CI) for asthma clinical encounters were estimated using a conditional logistic regression model per interquartile range (IQR) increase in short-term PM_2.5_ exposure in a case-crossover study design. Each child served as their own control allowing implicit control for any confounders that do not temporally vary over the short term, including possible indoor air pollutant exposures such as secondhand cigarette smoke and exposures from indoor wood burning. The ‘index date’ was the day each child was considered to be a case based on having an asthma clinical encounter, and the ‘referent date’ was when the child was not considered a case, and served as a self-matched control. We used a semi-symmetric bidirectional referent design minimizing seasonal confounding bias by using narrow referent time periods [35, 36]. Referent dates were the same day of the week as the index date to allow for more accurate exposure assignments since there are differences in PM_2.5_ emissions depending on the day of the week. To avoid overlap bias, referent days were randomly selected 7 days before or after the clinical event. For any observation where one of the possible referent days did not fall within our study period, we assigned an offset term of log (2) [37]. The analysis used short exposure lags of 0, 1, and 2 days before both the index date and the referent date to analyze asthma symptom exacerbation with exposure to PM_2.5_.

Changes in residency between clinical encounter and referent date were unlikely to be problematic because the index date and the referent date were only 7 days apart. For this same reason, differences in traffic characteristics on the same day-of-the-week were not controlled for as they were unlikely to change during that time. Variables that were controlled for, and included in the fully adjusted model, were those that had short-term changes including temperature, humidity, barometric pressure and whether a referent or index date occurred on a holiday (Memorial Day, Labor Day, Christmas Day, New Year’s Day, and Independence Day). Daily mean PM_2.5_ concentrations, temperature, humidity and barometric pressure were assigned to each study participant using ArcGIS. We assessed effect modification by stratifying on the following PELL data variables: season of clinical encounter, hospitalization frequency, insurance type, maternal education, previous bronchiolitis encounter, residential distance to major roadway, gestational age, birthweight, median income, breastfeeding status, cigarette use during pregnancy, maternal language preference and child’s gender. We present *p*-values for the interaction term added to the fully adjusted model between PM_2.5_ and each variable.

## Results

Among the 33,387 asthma cases 0–9 years of age, the majority were male (63.2%), had a gestational age ≥ 37 weeks (86.4%), normal birthweight (89.9%) and were not previously admitted for a bronchiolitis encounter (71.7%). For most mothers of asthma cases, this was not their first child (58.9%), the majority were non-smokers during pregnancy (88.2%), white (59.3%), English-speaking (87.5%), lived within 150 m of a roadway (53.2%), had a household income between $20,000–$70,000 (55.5%), did not use government paid health insurance (55.7%), had more than a high school education (51.1%), had initiated breastfeeding at the hospital following the birth of their child (72.3), and did not report drinking during their pregnancy (98.3%) Relatively few participants had missing characteristic data (0.1%–0.5%). (Table 1).

**Table 1:** Selected demographic characteristics among children 0–9 years of age with asthma clinical encounters

Characteristics	N (%)^a^
Child’s Sex	
Male	21,105 (63.2)
Female	12,282 (36.8)
Gestational Age	
Full term (> = 37 weeks)	28,863 (86.4)
Preterm (< 37 weeks)	4510 (13.5)
Missing	14 (0.1)
Birthweight	
Normal (> = 2500 g)	30,024 (89.9)
Low (< 2500 g)	3241 (9.7)
Missing	122 (0.4)
Previous Bronchiolitis Clinical Encounter	
Yes	9440 (28.3)
No	23,947 (71.7)
Frequency of Clinical Encounters	
Admitted once	18,272 (54.7)
Admitted more than once	15,115 (45.3)
Maternal Age	
< 20	3437 (10.3)
20–24	6916 (20.7)
25–29	7980 (23.9)
30–34	8851 (26.5)
35+	6203 (18.6)
Parity	
0	13,574 (40.7)
1	11,379 (34.1)
2+	8293 (24.8)
Missing	141 (0.4)
Maternal Education	
Less than High School	5597 (16.8)
High School	10,662 (31.9)
More than High School	17,064 (51.1)
Missing	64 (0.2)
Smoking during pregnancy	
Yes	3922 (11.7)
No	29,410 (88.2)
Missing	55 (0.2)
Alcohol during pregnancy	
Yes	510 (1.5)
No	32,827 (98.3)
Missing	50 (0.2)
Maternal Race/Ethnicity	
White	19,789 (59.3)
Black	4011 (12.0)
Hispanic	7099 (21.3)
Asian/Pacific Islander	1592 (4.8)
Other	861 (2.6)
Missing	35 (0.1)
Maternal Language Preference	
English	29,203 (87.5)
Spanish	2498 (7.5)
Portuguese	662 (2.0)
Other	240 (0.7)
Missing	138 (0.4)
Household Income	
< $20,000	2596 (7.8)
$20,000–$70,000	18,518 (55.5)
> = $70,000	12,273 (36.8)
Source of Payment	
Government Paid	14,660 (43.9)
Other	18,608 (55.7)
Missing	119 (0.4)
Breastfed initiation at hospital	
Yes	24,137 (72.3)
No	9098 (27.2)
Missing	152 (0.5)
Distance to Nearest Roadway	
< =150 m	17,749 (53.2)
> 150 m	15,638 (46.8)

^a^Percentages may not add up to 100% in some subgroups because of rounding

Approximately 10% of our study sample had missing PM_2.5_ concentrations due to possible cloud or snow cover when the satellite based model was unable to provide estimates for a given day. For lag 0, the IQR for PM_2.5_ was 5.9 μg/m^3^. The mean PM_2.5_ concentration was 8.8 μg/m^3^ and the median concentration was 7.8 μg/m^3^. Similar distributions were observed for lags 1 and 2.

No statistically significant associations were found between PM_2.5_ exposures and asthma clinical encounters for lags 0, 1, and 2 regardless of whether only primary and secondary diagnosis fields were used (Table 2).

**Table 2:** Odds ratios per IQR increase in PM_2.5_ and asthma clinical encounters

	Lag 0^a^		Lag 1^b^		Lag 2^c^	
	N	OR (95% CI)	N	OR (95% CI)	N	OR (95% CI)
Crude Model	29,781	1.00 (0.98, 1.02)	29,840	1.00 (0.98, 1.02)	29,828	1.00 (0.98, 1.01)
Adjusted Model^d^	28,777	0.99 (0.98, 1.01)	28,829	1.00 (0.98, 1.02)	28,830	0.98 (0.96, 1.00)
Primary/ Secondary Diagnosis only^d^	26,185	0.99 (0.97, 1.01)	26,230	1.00 (0.98, 1.02)	26,219	0.98 (0.96, 1.01)

^a^Lag 0 corresponds to a clinical encounter for the day of exposure

^b^Lag 1 corresponds to exposure a day prior to clinical encounter

^c^Lag 2 corresponds to exposure two days prior to clinical encounter

^d^Adjusted for lagged temperature, humidity, barometric pressure, and holiday indicator

Effect modification was investigated for lags 0, 1 and 2 by stratifying to identify potentially susceptible subgroups (Table 3). Differences in odds ratios were observed when stratifying on birthweight. The association between PM_2.5_ exposure and having an asthma clinical encounter was significantly different among children with birthweight less than 2500 g (g) compared to those greater than or equal to 2500 g across all lags (*p* < 0.05), with the strongest association observed among low birthweight (LBW) children for lag 1 (OR: 1.08; 95% CI: 1.01, 1.15) compared to normal birthweight (OR: 0.99; 95% CI: 0.97, 1.01) per IQR increase in PM_2.5_.

**Table 3:** Odds ratios per IQR increase in PM_2.5_ and asthma clinical encounters with stratified analysis

OR (95% Confidence Interval)^a^	Lag 0^b^	*P*-value^e^	Lag 1^c^	*P*-value^e^	Lag 2^d^	*P*-value^e^
Season of Clinical Encounter						
Warm	0.97 (0.94, 0.99)	0.01*	0.97 (0.94, 1.00)	0.04*	0.97 (0.94, 1.00)	0.34
Cold	1.02 (0.99, 1.05)		1.02 (0.99, 1.05)		1.00 (0.97, 1.03)	
Insurance Type						
Government Paid	1.00 (0.97, 1.03)	0.42	1.02 (0.99, 1.05)	0.06	1.00 (0.97, 1.04)	0.13
Other	0.98 (0.96, 1.01)		0.98 (0.95, 1.00)		0.97 (0.94, 0.99)	
Frequency of Clinical Encounters						
One time	1.00 (0.97, 1.03)	0.09	1.01 (0.98, 1.04)	0.26	0.98 (0.95, 1.00)	0.17
More than one	0.98 (0.95, 1.01)		0.98 (0.95, 1.02)		0.99 (0.96, 1.02)	
Maternal Education						
Less than HS	0.99 (0.95, 1.04)	0.33	1.00 (0.95, 1.05)	0.77	0.97 (0.93, 1.02)	0.36
High School	1.02 (0.98, 1.05)		1.01 (0.97, 1.05)		1.01 (0.97, 1.05)	
More than HS	0.98 (0.95, 1.00)		0.99 (0.96, 1.02)		0.97 (0.94, 1.00)	
Previous Bronchiolitis encounter						
Yes	1.00 (0.97, 1.03)	0.84	1.02 (0.98, 1.06)	0.37	1.01 (0.97, 1.05)	0.06
No	0.99 (0.97, 1.01)		0.99 (0.96, 1.01)		0.97 (0.95, 1.00)	
Residential Distance to Major Roadway						
< =150 m	1.00 (0.97, 1.04)	0.60	0.98 (0.95, 1.01)	0.05	0.99 (0.97, 1.02)	0.49
> 150 m	0.99 (0.96, 1.02)		1.02 (0.99, 1.05)		0.97 (0.94, 1.00)	
Gestational Age						
Preterm (< 37 weeks)	0.99 (0.94, 1.05)	0.97	1.03 (0.97, 1.09)	0.20	1.02 (0.97, 1.08)	0.07
Full Term (> = 37 weeks)	0.99 (0.97, 1.01)		0.99 (0.97, 1.02)		0.98 (0.96, 1.00)	
Birthweight						
Low (< 2500 g)	1.05(0.98, 1.12)	0.04*	1.08 (1.01, 1.15)	0.01*	1.06 (0.99, 1.13)	0.004*
Normal (≥2500 g)	0.99 (0.97, 1.01)		0.99 (0.97, 1.01)		0.97 (0.95, 0.99)	
Median Income						
< $20,000	0.96 (0.89, 1.03)	0.33	1.01 (0.94, 1.09)	0.80	1.00 (0.93, 1.07)	0.71
$20,000–$70,000	1.00 (0.97, 1.03)		0.99 (0.96, 1.02)		0.98 (0.95, 1.00)	
> $70,000	0.99 (0.96, 1.03)		1.01 (0.97, 1.04)		0.99 (0.96, 1.03)	
Breastfeeding Status						
Yes	0.98 (0.96, 1.01)	0.17	0.99 (0.97, 1.02)	0.57	0.98 (0.95, 1.00)	0.56
No	1.02 (0.98, 1.06)		1.01 (0.97, 1.05)		1.00 (0.96, 1.04)	
Smoking During Pregnancy						
Yes	1.01 (0.95, 1.07)	0.64	1.03 (0.97, 1.03)	0.20	0.99 (0.94, 1.05)	0.65
No	0.99 (0.97, 1.01)		0.99 (0.97, 1.02)		0.98 (0.96, 1.00)	
Maternal Language Preference						
English	0.99 (0.97, 1.01)	0.82	1.00 (0.98, 1.02)	0.40	0.98 (0.96, 1.00)	0.86
Other	1.00 (0.95, 1.06)		0.98 (0.92, 1.04)		0.99 (0.93, 1.05)	
Race						
White	1.00 (0.98, 1.03)	0.32	0.99 (0.97, 1.02)	0.59	0.98 (0.95, 1.00)	0.85
Other	0.98 (0.95, 1.01)		1.00 (0.97, 1.04)		0.99 (0.96, 1.02)	
Gender						
Male	1.00 (0.97, 1.02)	0.52	0.98 (0.96, 1.01)	0.19	0.98 (0.95, 1.01)	0.88
Female	0.99 (0.95, 1.02)		1.02 (0.98, 1.06)		0.99 (0.96, 1.03)	

^a^Adjusted for lagged temperature, humidity, barometric pressure, and holiday indicator

^b^Lag 0 corresponds to a clinical encounter for the day of exposure

^c^Lag 1 corresponds to exposure a day prior to clinical encounter

^d^Lag 2 corresponds to exposure two days prior to clinical encounter

^e^*p*-value presented is from the interaction term of each risk factor and PM_2.5_ added to the fully adjusted model

*statistical significance (*p* < 0.05) for interaction term

Per IQR increase in PM_2.5_, non-significant increases in the odds of having an asthma clinical encounter were observed for preterm children for lag 1 (OR: 1.03; 95% CI: 0.97, 1.09) and lag 2 (OR: 1.02; 95% CI: 0.97, 1.08) compared to full-term children (lag 1 OR: 0.99; 95% CI: 0.97, 1.02; lag 2 OR: 0.98; 95% CI: 0.96, 1.00). The odds of an asthma clinical encounter were significantly higher (*p* < 0.05) among children who experienced a clinical encounter in the cold season (OR: 1.02; 95% CI: 0.99, 1.05) compared to the warm season (OR: 0.97, 95% CI: 0.94, 0.99) per IQR increase in PM_2.5_ for lag 0 with similar results for lag 1. Among children whose insurance type was not paid through the government a protective effect was observed for lag 2 (OR: 0.97; 95% CI: 0.94, 0.99) compared to those who had government paid insurance (OR: 1.00; 95% CI: 0.98, 1.04) per IQR increase in PM_2.5_. Similar results were observed for lags 0 and 1, although not significant (Table 3).

Results for the analysis of children 5–9 years of age are presented in Additional file 1: Tables S1-S3. Characteristics among the 6195 children and mothers in the 5–9 years of age group were consistent with those 0–9 years of age (Additional file 1: Table S1). Again, asthma clinical encounters were not associated with PM_2.5_ exposures for lags 0, 1, and 2 regardless of whether only primary and secondary diagnosis fields were used (Additional file 1: Table S2). We observed negative associations for lag 0 when stratifying on gestational age, maternal race, residential distance to roadway, median income, breastfeeding initiation at hospital, and child’s gender. Per IQR increase in PM_2.5,_ preterm children (< 37 weeks) had significantly lower odds of having an asthma clinical encounter (OR: 0.81; 95% CI: 0.71, 0.92) compared to those were born full-term (> = 37 weeks) (OR: 0.98; 95% CI: 0.94, 1.03). Children who were born to mothers whose race was other than white had significantly decreased odds of having an asthma clinical encounter (OR: 0.91; 95% CI: 0.84, 0.98) per IQR increase in PM_2.5_ compared to those born to white mothers (OR: 0.99; 95% CI: 0.93, 1.05). Among children who lived within 150 m from a roadway we did not observe any association per IQR increase in PM_2.5_ (OR: 1.00; 95% CI: 0.94, 1.06), whereas a protective effect was observed among those who lived greater than 150 m from a roadway (OR: 0.92; 95% CI: 0.86, 0.98). Similar results were observed across lags 1 and 2, although not significant. Per IQR increase in PM_2.5_, those with an annual household median income between $20,000–$70,000 had decreased odds of an asthma clinical encounter (OR: 0.92; 95% CI: 0.86, 0.98) compared to those whose median income was greater than $70,000 (OR: 1.01; 95% CI: 0.94, 1.09) and those who were less than $20,000 (OR: 1.02; 95% CI: 0.87, 1.19). Odds of having a clinical encounter per IQR increase in PM_2.5_ were lower for female children (OR: 0.92; 95% CI: 0.85, 0.99) compared to male children (OR: 0.98; 95% CI: 0.93, 1.04). Lastly, children of mothers who initiated breastfeeding at the hospital following delivery were observed to have a protective effect against asthma clinical encounters per IQR increase in PM_2.5_ (OR: 0.93; 95% CI: 0.88, 0.98) compared to those who did not initiate breast feeding (OR: 1.03; 95% CI: 0.95, 1.12) (Additional file 1: Table S3).

## Discussion

Several studies have suggested increased asthma exacerbation with short-term PM_2.5_ exposure [25] but little has been reported on the subgroups of children with asthmatic symptoms that may be most susceptible to the effects of PM_2.5._ Further, a broad range of PM_2.5_ concentrations in the various study locations have been reported [27]. In our study area, we report lower levels of PM_2.5_ with a median value of 7.8 μg/m^3^, however it has been suggested that asthma exacerbations may begin to increase at PM_2.5_ levels as low as 4.00–7.06 μg/m^3^ [38]. Our study identified sensitive subgroups that may be more vulnerable to low levels of PM_2.5_-induced asthma exacerbation resulting in a clinical encounter. LBW has been associated with a range of health effects in children including respiratory infections, lowered lung function and asthma [39–41]. LBW children have also displayed increased asthma symptoms related to air pollutants [42, 43]. When stratifying on birthweight, we saw increased odds of having an asthma clinical encounter across all lags, and statistically significant differences in the effect of PM_2.5_ on asthma clinical encounters for LBW children compared to normal birthweight. This demonstrates that birthweight may play a role in immune development and subsequent asthma development.

In addition to LBW, the effect of PM_2.5_ on asthma clinical encounters was significantly different in the cold season compared to the warm season (*p* < 0.05). Previous studies have identified the effects of air pollution in both warm and cool seasons to be associated with pediatric asthma attacks [21–24].

In this study we did not find smoking during pregnancy to be a significant effect modifier; however, we did observe increased odds ratios among women who smoked during pregnancy compared to those who did not across lags 1 and 2. Several studies have shown that maternal smoking during pregnancy is a modifiable risk factor for asthma [44]. The lack of effect modification by smoking in our analyses may be due to the relatively small number of women who reported smoking during pregnancy (*n* = 3922) and the under-reporting of smoking by pregnant women, causing misclassification of that variable.

Higher rates of asthma have been reported along major roadways [17] and exposure to traffic-related air pollutants have been associated with an increased risk of asthma hospitalizations [45, 46]. We found non-significant and inconsistent results within our data among children who lived within 150 m of roadways. In our analysis with children 5 and older, we found non-significant increases in odds ratios among children who lived within 150 m of a major roadway compared to those who lived greater than 150 m for lag 2 per IQR increase in PM_2.5_, and a statistically significant protective effect among those who lived greater than 150 m per IQR increase in PM_2.5_ for lag 0 (Additional file 1: Table S3). In our study, we specifically determined distance to major roadway from the household residence. However, studies have identified near roadway exposures at school may play a role in asthma outcomes as well [47].

Because asthma is best diagnosed at age 5 or older, we performed a secondary analysis restricting our cases those who had a clinical encounter at 5 years of age and older. We chose not to use these cases as our primary analysis because our case definition for asthma or wheeze included children who had the most severe symptoms leading them to seek care in the emergency department, and the majority of our cases were under the age of 5. Among those children who had a clinical encounter at age 5 or older, the association between PM_2.5_ exposure and having an asthma clinical encounter was significantly different among preterm children compared to the full-term children for lag 0 (*p* < 0.05). We observed significantly decreased odds of asthma clinical encounters among pre-term children (OR: 0.81; 95% CI: 0.71, 0.92) compared to full-term children (OR: 0.98; 95% CI: 0.94, 1.03) per IQR increase in PM_2.5_ (Additional file 1: Table S3). Preterm children may have other health issues that are typically treated more often in outpatient clinical settings, and therefore may have their asthma well-controlled and are seen less often in the emergency department. In addition, older children may have already been diagnosed with asthma in an outpatient clinical setting, or may have been seen in the emergency department for asthma or wheeze prior to age 5.

Our study tested several susceptible subgroups, and although we did not formally test for multiple comparisons, our significant findings exceeded the 5% type 1 error rate, or false positive rate, that we would have expected under the null hypothesis of no associations. Of the 42 tests between effect modifiers and PM_2.5_ exposure among children 0–9 years of age, there were 5 (11.9%) significant interactions found (Table 3). In the analysis with children 5–9 years of age, there was only 1 significant interaction found between PM_2.5_ and gestational age and therefore was within the 5% type 1 error rate and may have been an expected finding due to chance (Additional file 1: Table S3).

Our study design allowed our cases to serve as self-matched controls which provided several strengths. We did not need to control for modifiable asthma risk factors such as secondhand smoke. We were able to adjust for short-term meteorological changes that affect PM_2.5_ concentrations. Another major strength of this study was our ability to use satellite-based measurements to obtain estimates of PM_2.5_ at a 4-km resolution instead of relying on data from a relatively small number of fixed monitoring stations. Using short-term lags was reasonable since we would expect parents to seek medical attention for poorly controlled asthma or exacerbation of asthma or wheeze within the same day, or a day or two after symptoms begin. Another strength of this study was that the PELL data afforded both the large sample size and the record linkage necessary to exclude children who moved between their time of birth and clinical encounter. Exposure was assigned based on residential address so using the street-level birth address and matching that to the zip code at the time of their clinical encounter reduced exposure misclassification. Demographic characteristics among the subgroup that moved (including race, income, and maternal education) were consistent with the characteristics of those that did not move.

Although one advantage of using a case-crossover design is that there is no confounding by time invariant factors, one limitation is that the study design doesn’t allow for independent estimation of the effects for time invariant characteristics. Another limitation of this study was that our cases included only children who had more severe or poorly controlled asthma with severe enough symptoms to seek clinical care, and did not include children who are seen in outpatient clinical settings. Our study strictly focused on PM_2.5_ exposure, however there has been accumulating literature focusing on traffic-related air pollutants (TRAP) as a whole instead of the independent effects of individual air pollutants [48]. Although we used distance to major roadway measurements to account for traffic-related air pollutants, further investigation of other air pollution components may be warranted.

## Conclusions

Our study identified susceptible subgroups of children that may have increased risk for asthma clinical encounters resulting from lower-level PM_2.5_ exposure. We found that LBW children had increased odds of an asthma or wheeze clinical encounter across all lags. Birthweight was a significant effect modifier at α = 0.05. We also found the season of clinical encounter to be a significant effect modifier for lags 0 and 1. This study suggests LBW children may be more susceptible to the effects of PM_2.5_. We did not find strong evidence of PM_2.5_ exposure and increased risk of asthma or wheeze clinical encounters among other subgroups.

## Additional file

Additional file 1: **Table S1.** Selected demographic characteristics among children 5–9 years of age with asthma clinical encounters. **Table S2.** Odds ratios for a 5 μg/m^3^ increase in PM_2.5_ and asthma clinical encounters. **Table S3.** Odds ratios for a 5 μg/m^3^ increase in PM_2.5_ and asthma clinical encounters with stratified analysis. (DOCX 43 kb)

## Abbreviations

CI: Confidence interval
g: grams
ICD-9: International Classification of Diseases, Ninth Revision
IQR: interquartile range
LBW: Low birthweight
MA: Massachusetts
OR: Odds ratio
PELL: Pregnancy to Early Life Longitudinal
PM_2.5_: Fine particulate matter less than 2.5 μm
