# Correction to: Early-life exposure to PM2.5 and risk of acute asthma clinical encounters among children in Massachusetts: a case-crossover analysis

**DOI:** 10.1186/s12940-018-0371-4

**Published:** 2018-03-06

**Authors:** Roxana Khalili, Scott M. Bartell, Xuefei Hu, Yang Liu, Howard H. Chang, Candice Belanoff, Matthew J. Strickland, Verónica M. Vieira

**Affiliations:** 1 Environmental Health Sciences Graduate Program, Susan and Henry Samueli College of Health Sciences, University of California, Irvine, CA USA; 2 Program in Public Health, Susan and Henry Samueli College of Health Sciences, University of California, Irvine, 653 E. Peltason Dr., AIRB 2042, Irvine, CA 92697-3957 USA; 3 Department of Statistics, Donald Bren School of Information and Computer Sciences, University of California, Irvine, CA USA; 4 Department of Epidemiology, School of Medicine, Susan and Henry Samueli College of Health Sciences, University of California, Irvine, CA USA; 5 Department of Environmental Health, Emory University Rollins School of Public Health, Atlanta, GA USA; 6 Department of Community Health Sciences, Boston University School of Public Health, Boston, MA USA; 7 School of Community Health Sciences, University of Nevada, Reno, NV USA

## Correction

After publication of the article [1], it was brought to our attention that a number in Table 1 is incorrect. The table quotes the number of children with Maternal Language Preference listed as “Other” as being “240 (0.7%)”. This should in fact be “886 (2.7%)”.

## References

[1] Khalili R, Bartell S, Hu X, Liu Y, Chang H, Belanoff C (2018). Early-life exposure to PM2.5 and risk of acute asthma clinical encounters among children in Massachusetts: a case-crossover analysis. Environ Health.

