# Supplementary material for: Early-life exposure to PM2.5 and risk of acute asthma clinical encounters among children in Massachusetts: a case-crossover analysis
Source: Environ Health. 2018 Feb 21;17:20. doi: 10.1186/s12940-018-0361-6 (PMC5822480; doi:10.1186/s12940-018-0361-6)
Supplement: Supplementary file 1 — Table S1. Selected demographic characteristics among children 5–9 years of age with asthma clinical encounters. Table S2. Odds ratios for a 5 μg/m3 increase in PM2.5 and asthma clinical encounters. Table S3. Odds ratios for a 5 μg/m3 increase in PM2.5 and asthma clinical encounters with stratified analysis. (DOCX 43 kb) [file 12940_2018_361_MOESM1_ESM.docx]

**Supplemental Material**

Early-life Exposure to PM_2.5_ and Risk of Acute Asthma Clinical Encounters among Children in Massachusetts: A Case-Crossover Analysis

Roxana Khalili, Scott M. Bartell, Xuefei Hu, Yang Liu, Howard H. Chang, Candice Belanoff, Matthew J. Strickland, Verónica M. Vieira

**Table of Contents**

Table S1. Selected demographic characteristics among children 5-9 years of age with asthma clinical encounters

Table S2. Odds ratios per IQRincrease in PM_2.5_ and asthma clinical encounters

Table S3. Odds ratios per IQR increase in PM_2.5_ and asthma clinical encounters with stratified analysis

and p-values for interaction term between each risk factor and PM_2.5_ added to the fully adjusted model

Table S1. Selected demographic characteristics among children 5-9 years of age with asthma clinical encounters

| Characteristics | N (%)^a^ |
| --- | --- |
| **Child’s Sex** |  |
| Male | 3889 (62.8) |
| Female | 2306 (37.2) |
| **Gestational Age** |  |
| Full term (>=37 weeks) | 5434 (88.2) |
| Preterm (<37 weeks) | 758 (12.2) |
| Missing | 3 (0.05) |
| **Birthweight** |  |
| Normal (>=2500 g) | 5667 (91.5) |
| Low (<2500 g) | 486 (7.8) |
| Missing | 42 (0.7) |
| **Previous Bronchiolitis Clinical Encounter** |  |
| Yes | 1236 (20.0) |
| No | 4959 (80.0) |
| **Frequency of Clinical Encounters** |  |
| Admitted once | 2542 (41.0) |
| Admitted more than once | 3653 (59.0) |
| **Parity** |  |
| 0 | 2675 (43.2) |
| 1 | 2060 (33.3) |
| 2+ | 1418 (22.9) |
| Missing | 42 (0.7) |
| **Maternal Education** |  |
| Less than High School | 911 (14.7) |
| High School | 1971 (31.8) |
| More than High School | 3296 (53.2) |
| Missing | 17 (0.3) |
| **Smoking during pregnancy** |  |
| Yes | 678 (10.9) |
| No | 5501 (88.8) |
| Missing | 16 (0.2) |
| **Alcohol during pregnancy** |  |
| Yes | 97 (1.6) |
| No | 6083 (98.2) |
| Missing | 15 (0.2) |
| **Maternal Race/Ethnicity** |  |
| White | 3902 (63.0) |
| Black | 650 (10.5) |
| Hispanic | 1179 (19.0) |
| Asian/Pacific Islander | 298 (4.8) |
| Other | 157 (2.5) |
| Missing | 9 (0.1) |
| **Maternal Language Preference** |  |
| English | 5426 (87.6) |
| Spanish | 437 (7.0) |
| Portuguese | 107 (1.7) |
| Other | 200 (3.2) |
| Missing | 25 (0.4) |
| **Household Income** |  |
| <$20000 | 430 (6.9) |
| $20000-$70000 | 3263 (52.7) |
| >=$70000 | 2492 (40.2) |
| Missing | 10 (0.2) |
| **Source of Payment** |  |
| Government Paid | 2325 (37.5) |
| Other | 3829 (61.8) |
| Missing | 41 (0.7) |
| **Breastfed initiation at hospital** |  |
| Yes | 4395 (70.9) |
| No | 1750 (28.2) |
| Missing | 50 (0.8) |
| **Distance to Road** |  |
| <=150 meters | 3244 (52.4) |
| >150 meters | 2951 (47.6) |

^a^Percentages may not add up to 100% in some subgroups because of rounding

Table S2. Odds ratios per IQR increase in PM_2.5_ and asthma clinical encounters

|  | **Lag 0^a^** | | **Lag 1^b^** | | **Lag 2^c^** | |
| --- | --- | --- | --- | --- | --- | --- |
|  | **N** | **OR (95% CI)** | **N** | **OR (95% CI)** | **N** | **OR (95% CI)** |
| **Crude**  **Model** | 5607 | 0.97 (0.93, 1.02) | 5627 | 1.01 (0.97, 1.06) | 5640 | 1.00 (0.96, 1.04) |
| **Adjusted**  **Model^d^** | 5593 | 0.96 (0.92, 1.00) | 5607 | 1.01 (0.96, 1.05) | 5624 | 1.00 (0.95, 1.04) |
| **Primary/**  **Secondary Diagnosis ^d^** | 5064 | 0.96 (0.92, 1.01) | 5077 | 1.01 (0.96, 1.06) | 5088 | 0.99 (0.95, 1.04) |

^a^Lag 0 corresponds to a clinical encounter for the day of exposure

^b^Lag 1 corresponds to exposure a day prior to clinical encounter

^c^Lag 2 corresponds to exposure two days prior to clinical encounter

^d^Adjusted for lagged temperature, humidity, barometric pressure, and holiday indicator

Table S3. Odds ratios for per IQRincrease in PM_2.5_ and asthma clinical encounters with stratified analysis

| **OR (95% Confidence Interval)^a^** | **Lag 0^b^** | P-value^e^ | **Lag 1^c^** | P-value^e^ | **Lag 2^d^** | P-value^e^ |
| --- | --- | --- | --- | --- | --- | --- |
| **Season of Clinical Encounter** |  |  |  |  |  |  |
| Warm | 0.94 (0.89, 1.00) | 0.32 | 1.00 (0.94, 1.07) | 0.96 | 1.00 (0.93, 1.05) | 0.63 |
| Cold | 0.97 (0.90, 1.05) |  | 1.02 (0.95, 1.09) |  | 1.01 (0.94, 1.09) |  |
| **Insurance Type** |  |  |  |  |  |  |
| Government Paid | 0.95 (0.88, 1.02) | 0.72 | 1.02 (0.94, 1.09) | 0.54 | 0.97 (0.90, 1.05) | 0.45 |
| Other | 0.97 (0.91, 1.03) |  | 0.99 (0.94, 1.05) |  | 1.01 (0.95, 1.07) |  |
| **Frequency of Clinical Encounters** |  |  |  |  |  |  |
| One time | 0.95 (0.89, 1.03) | 0.49 | 0.96 (0.89, 1.04) | 0.43 | 0.96 (0.89, 1.03) | 0.35 |
| More than one time | 0.96 (0.91, 1.02) |  | 1.03 (0.97, 1.09) |  | 1.02 (0.96, 1.08) |  |
| **Education** |  |  |  |  |  |  |
| Less than High School | 0.94 (0.84, 1.06) | 0.72 | 0.95 (0.84, 1.07) | 0.73 | 0.92 (0.82, 1.04) | 0.20 |
| High School | 0.99 (0.91, 1.07) |  | 1.01 (0.93, 1.09) |  | 0.96 (0.89, 1.04) |  |
| More than High School | 0.94 (0.88, 1.01) |  | 1.02 (0.96, 1.09) |  | 1.04 (0.97, 1.11) |  |
| **Previous Bronchiolitis encounter** |  |  |  |  |  |  |
| Yes | 0.98 (0.88, 1.08) | 0.59 | 1.02 (0.92, 1.12) | 0.77 | 0.99 (0.89, 1.10) | 0.94 |
| No | 0.96 (0.91, 1.01) |  | 1.00(0.95, 1.06) |  | 1.00 (0.95, 1.05) |  |
| **Residential Distance to Major Roadway** |  |  |  |  |  |  |
| <=150 meters | 1.00 (0.94, 1.06) | 0.09 | 1.05 (0.99, 1.12) | 0.11 | 1.00 (0.94, 1.07) | 0.99 |
| >150 meters | 0.92 (0.86, 0.98) |  | 0.96 (0.89, 1.02) |  | 0.99 (0.93, 1.06) |  |
| **Gestational Age** |  |  |  |  |  |  |
| Preterm  (<37 weeks) | 0.81 (0.71, 0.92) | 0.01* | 1.01 (0.89, 1.14) | 0.95 | 1.04 (0.91, 1.18) | 0.31 |
| Full Term (>=37 weeks) | 0.98 (0.94, 1.03) |  | 1.01 (0.96, 1.06) |  | 0.99 (0.94, 1.04) |  |
| **Birthweight** |  |  |  |  |  |  |
| Low (<2500 g) | 0.91 (0.78, 1.07) | 0.48 | 1.06 (0.91, 1.25) | 0.47 | 0.96 (0.82, 1.13) | 0.76 |
| Normal (>=2500g) | 0.96 (0.92, 1.01) |  | 1.00 (0.95, 1.05) |  | 1.00 (0.95, 1.05) |  |
| **Median Income** |  |  |  |  |  |  |
| <$20000 | 1.02 (0.87, 1.19) | 0.21 | 1.01(0.85, 1.19) | 0.82 | 1.08 (0.90, 1.30) | 0.56 |
| $20000-$70000 | 0.92 (0.86, 0.98) |  | 0.98 (0.92, 1.05) |  | 0.98 (0.92, 1.05) |  |
| >$70000 | 1.01 (0.94, 1.09) |  | 1.03 (0.96, 1.11) |  | 1.00 (0.93, 1.07) |  |
| **Breastfeeding Status** |  |  |  |  |  |  |
| Yes | 0.93 (0.88, 0.98) | 0.06 | 1.01 (0.96, 1.07) | 0.40 | 0.99 (0.97, 1.02) | 0.75 |
| No | 1.03 (0.95, 1.12) |  | 0.98 (0.90, 1.07) |  | 0.99 (0.91, 1.08) |  |
| **Smoking During Pregnancy** |  |  |  |  |  |  |
| Yes | 1.01 (0.89, 1.15) | 0.75 | 0.99 (0.87, 1.12) | 0.64 | 0.93 (0.81, 1.07) | 0.14 |
| No | 0.95 (0.91, 1.00) |  | 1.01 (0.96, 1.06) |  | 1.00 (0.96, 1.05) |  |
| **Maternal Language Preference** |  |  |  |  |  |  |
| English | 0.97 (0.92, 1.02) | 0.12 | 1.00 (0.95, 1.05) | 0.36 | 1.00 (0.95, 1.05) | 0.84 |
| Other | 0.88 (0.77, 1.00) |  | 1.07 (0.93, 1.22) |  | 0.99 (0.87, 1.13) |  |
| **Race** |  |  |  |  |  |  |
| White | 0.99 (0.93, 1.05) | 0.05 | 1.02 (0.96, 1.08) | 0.84 | 0.99 (0.94, 1.05) | 0.69 |
| Other | 0.91 (0.84, 0.98) |  | 0.98 (0.91, 1.06) |  | 1.01 (0.93, 1.09) |  |
| **Gender** |  |  |  |  |  |  |
| Male | 0.98 (0.93, 1.04) | 0.11 | 1.02 (0.96, 1.08) | 0.61 | 0.97 (0.92, 1.03) | 0.45 |
| Female | 0.92 (0.85, 0.99) |  | 1.00 (0.92, 1.07) |  | 1.03 (0.96, 1.11) |  |

^a^Adjusted for lagged temperature, humidity, barometric pressure, and holiday indicator

^b^Lag 0 corresponds to a clinical encounter for the day of exposure

^c^Lag 1 corresponds to exposure a day prior to clinical encounter

^d^Lag 2 corresponds to exposure two days prior to clinical encounter

^e^p-value presented is from the interaction term of each risk factor and PM_2.5_ added to the fully adjusted model

*statistical significance (p<0.05) for interaction term
